# Did COVID and Low Sodium Break My Heart?

**DOI:** 10.7759/cureus.105673

**Published:** 2026-03-22

**Authors:** Vishal Akula, Edward Tubberville, Mhorys Pickmans

**Affiliations:** 1 Internal Medicine, Mary Washington Healthcare, Fredericksburg, USA

**Keywords:** acute hyponatremia, covid 19, covid-induced, drug-induced hyponatremia, takotsubo cardiomyopathy (ttc)

## Abstract

Takotsubo cardiomyopathy is a transient cardiomyopathy that affects a substantial number of patients each year and has become increasingly recognized in clinical practice. Although its exact pathophysiology remains incompletely understood, the most widely accepted hypothesis attributes the condition to catecholamine-mediated myocardial stunning in response to emotional or physiological stress. Reported triggers are diverse and include medication exposure, infections, and electrolyte abnormalities. In this report, we present a patient diagnosed with Takotsubo cardiomyopathy in whom the precipitating stressor was initially unclear. On presentation, the patient was found to be COVID-19 positive and had severe hyponatremia, both of which were thought to have contributed to the development of her cardiomyopathy. We review the diagnostic approach used in this case and discuss the various diagnostic criteria currently available for Takotsubo cardiomyopathy. Additionally, we highlight aspects of these criteria that may not be universally required for diagnosis, with implications for reducing unnecessary testing, overall cost of care, and hospital expenditures.

## Introduction

Takotsubo cardiomyopathy is a transient syndrome of acute left ventricular dysfunction that closely mimics acute coronary syndrome (ACS) in its clinical presentation, electrocardiographic findings, and cardiac biomarker elevation. It accounts for approximately 1-4% of patients evaluated for suspected ACS, frequently complicating the initial diagnostic assessment [1]. It is characterized by reversible regional wall motion abnormalities that extend beyond a single coronary artery distribution in the absence of obstructive coronary artery disease [2].

Although the precise pathophysiology remains incompletely understood, the most widely accepted mechanism involves catecholamine-mediated myocardial stunning triggered by emotional or physiological stress, with consistently elevated circulating catecholamine levels demonstrated in affected patients [2,3]. Excess sympathetic activation results in β-adrenergic receptor overstimulation, intracellular calcium overload, and impaired myocardial energy utilization, ultimately leading to transient contractile dysfunction. Regional variations in β-adrenergic receptor density, particularly within the apical myocardium, may explain the characteristic distribution of wall motion abnormalities. Furthermore, catecholamine excess can alter ventricular myocyte action potentials, prolong repolarization, and contribute to the electrocardiographic changes frequently observed in Takotsubo cardiomyopathy [2].

Initially described in postmenopausal women following emotional stress, Takotsubo cardiomyopathy is now recognized to occur in response to a broad range of physical stressors. These include, but are not limited to, neurologic disorders, surgical procedures, pain, metabolic derangements, and infectious processes, all of which may precipitate myocardial stunning through neurohormonal activation [2]. Notably, COVID-19 infection has emerged as an increasingly recognized physiologic trigger, particularly among older adults with multiple comorbidities [2].

Because Takotsubo cardiomyopathy closely resembles other conditions, such as ACS or myocarditis, clinicians often pursue additional diagnostic evaluation, including advanced imaging modalities such as cardiac magnetic resonance imaging (cMRI). However, validated diagnostic criteria allow for a confident diagnosis in many cases without the need for cMRI. The Revised Mayo Clinic Criteria emphasize transient ventricular dysfunction, absence of obstructive coronary disease, and exclusion of alternative diagnoses, such as pheochromocytoma or myocarditis, based on clinical, angiographic, and echocardiographic findings [2]. Similarly, the InterTAK Diagnostic Score incorporates seven readily available clinical variables and demonstrates a specificity of 95% for diagnosing Takotsubo cardiomyopathy at a cutoff score of ≥50 points [4]. Thoughtful application of these criteria can streamline care and reduce healthcare expenditures without compromising diagnostic accuracy.

We present a case of Takotsubo cardiomyopathy in an elderly woman with concurrent COVID-19 infection and profound hyponatremia, likely related to an increased hydrochlorothiazide (HCTZ) dose contributing to renal sodium loss, in addition to gastrointestinal losses from vomiting. This case illustrates how systematic application of established diagnostic criteria can expedite diagnosis, guide appropriate management, and obviate the need for advanced imaging in the setting of multiple physiologic stressors accompanied by characteristic cardiac imaging findings.

## Case presentation

An 84-year-old woman with a history of hypertension and hyperlipidemia presented with one week of fever, chills, fatigue, nausea, and decreased appetite. Her primary care provider had recently increased her HCTZ dose to 25 mg daily. She was also taking 5 mg of amlodipine and atorvastatin 20 mg daily. On admission, she was hemodynamically stable with a normal mental status and was alert and oriented, without evidence of encephalopathy. Physical examination revealed no signs of volume overload, including the absence of jugular venous distention, clear lung fields on auscultation, and no peripheral edema.

**Figure 1:**
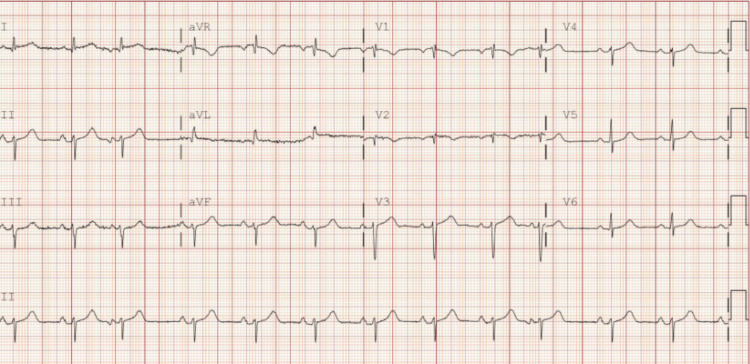
EKG EKG: electrocardiogram T-wave inversions in V1-V2 without ST elevation

Laboratory studies are presented in Tables 1-3. The complete blood count (CBC) was unremarkable, with no evidence of anemia, leukocytosis, leukopenia, or thrombocytopenia. The chest radiograph showed no acute cardiopulmonary process, including no consolidations, pleural effusions, or pulmonary edema. The patient’s electrocardiogram (EKG) demonstrated T-wave inversions in leads V1-V2 (Figure 1). There were no prior EKGs available in the medical record for comparison.

**Table 1:** Comprehensive metabolic panel AST: aspartate aminotransferase; ALT: alanine aminotransferase; eGFR: estimated glomerular filtration rate Laboratory values are presented with corresponding reference ranges. Abnormal results are indicated as elevated (↑) or decreased (↓) relative to standard laboratory reference ranges. eGFR is reported as >60 mL/min/1.73 m² per institutional laboratory reporting conventions

Laboratory test	Result	Reference range
Glucose	148 mg/dL (high)	70-99 mg/dL
Blood urea nitrogen (BUN)	10 mg/dL	7-20 mg/dL
Creatinine	0.5 mg/dL (low)	0.6-1.3 mg/dL
Sodium	119 mmol/L (low)	135-145 mmol/L
Potassium	3.6 mmol/L	3.5-5.1 mmol/L
Chloride	82 mmol/L (low)	98-107 mmol/L
CO₂ (bicarbonate)	27 mmol/L	22-29 mmol/L
Calcium	9.8 mg/dL	8.6-10.2 mg/dL
Total protein	7.1 g/dL	6.0-8.3 g/dL
Albumin	4.5 g/dL	3.5-5.0 g/dL
AST	51 U/L (high)	10-40 U/L
ALT	40 U/L	7-56 U/L
Alkaline phosphatase	49 U/L	40-129 U/L
Total bilirubin	0.7 mg/dL	0.2-1.2 mg/dL
eGFR	>60 mL/min/1.73 m²	≥60 mL/min/1.73 m²
Anion gap	10	8-16

**Table 2:** Hyponatremia studies Serum and urine laboratory values are presented with corresponding reference ranges. Abnormal values are indicated as decreased (low) or elevated (high). Urine osmolality is dependent on hydration status; values >100 mOsm/kg generally reflect impaired free water excretion

Laboratory test	Result	Reference range
Serum osmolality	254 mOsm/kg (Low)	275-295 mOsm/kg
Urine osmolality	584 mOsm/kg (High)	50-1,200 mOsm/kg
Urine sodium	19 mmol/L (Low)	20-40 mmol/L

**Table 3:** Cardiac biomarkers and COVID testing

Laboratory test	Result	Reference range
Troponin	1.96 ng/mL (High)	<0.04 ng/mL
B-type natriuretic peptide (BNP)	4,500 pg/mL (High)	<100 pg/mL
SARS-CoV-2 PCR	Positive	Negative

The patient was admitted for severe hyponatremia and a suspected non-ST-segment elevation myocardial infarction (NSTEMI). Her baseline serum sodium levels had previously ranged between 138 and 142 mEq/L. Gentle intravenous fluid therapy was initiated for the correction of hyponatremia. Concurrently, she received an aspirin loading dose and was started on a heparin infusion for management of the suspected NSTEMI.

Transthoracic echocardiography revealed newly reduced left ventricular systolic function, with an ejection fraction of 30-35%. The basal segments demonstrated preserved contractility, whereas the mid and apical segments were akinetic, findings consistent with a stress-induced cardiomyopathy pattern. These findings are illustrated in Video 1, which highlights the differences in myocardial motion during systole and diastole. Notably, this was the patient’s first echocardiographic evaluation.

**Video 1:** 4 Chamber view of normal contraction of left ventricle basal segments, with akinetic mid and apical segments

**Figure 2:**
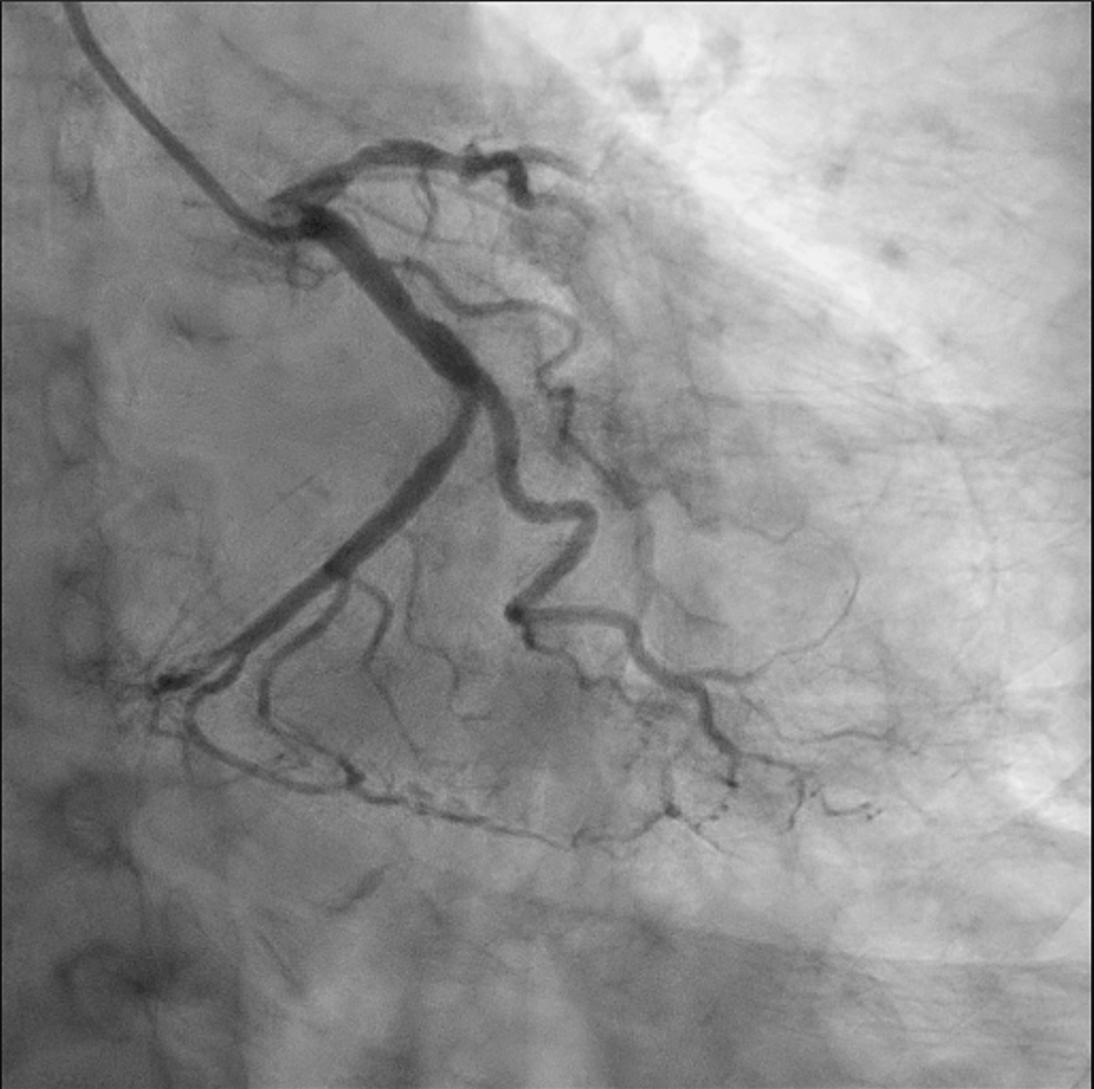
Right anterior caudal view of the left circumflex artery

**Figure 3:**
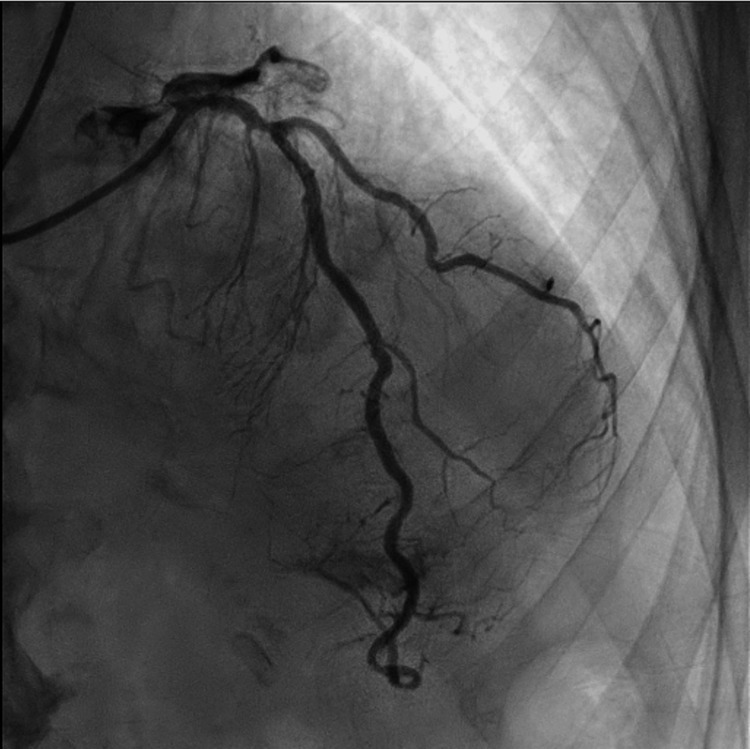
Left oblique anterior cranial view of the left anterior descending artery

**Figure 4:**
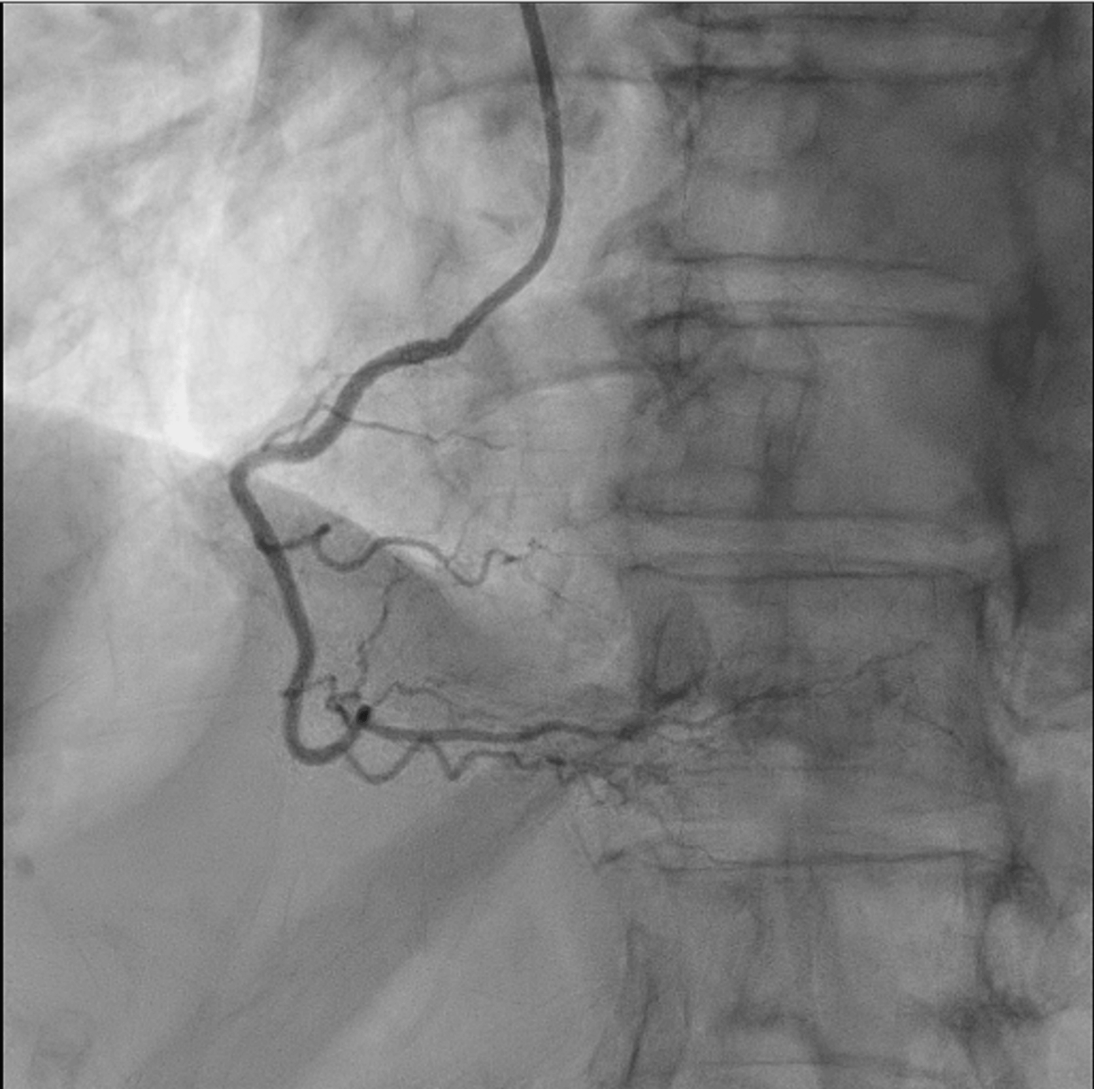
Left anterior oblique view of the right coronary artery

Following normalization of serum sodium levels, coronary angiography demonstrated mild, nonobstructive coronary artery disease, along with dyskinesis of the mid-to-distal anterior segments, thereby confirming the diagnosis of Takotsubo cardiomyopathy. Representative coronary angiographic images are provided in Figures 2-4.

Following confirmation of the diagnosis of Takotsubo cardiomyopathy, further evaluation of the patient’s mental health was undertaken. Both the patient and her husband reported that she had been experiencing increased stress recently and had not been adequately caring for herself. She described multiple recent stressors related to her health and family, which may have served as contributing emotional triggers for her stress cardiomyopathy.

Following electrolyte stabilization, she was started on losartan 25 mg daily for hypertension and stress cardiomyopathy. She was discharged on dual antiplatelet therapy with aspirin 81 mg and clopidogrel 75 mg daily. HCTZ was discontinued due to hyponatremia, and she was advised to have a close outpatient follow-up with her primary care provider and cardiologist to monitor ventricular recovery. 

## Discussion

Takotsubo cardiomyopathy is a transient, stress-induced left ventricular dysfunction typically precipitated by emotional or physiological stressors. Reported triggers include bereavement, trauma, acute medical illness, stimulant exposure, and metabolic disturbances. It accounts for 1-2% of patients initially evaluated for suspected ACS. The most widely accepted hypothesis is that elevated circulating catecholamine levels in response to a stressor underlie this syndrome, a finding repeatedly demonstrated in patients with Takotsubo cardiomyopathy. Excess stimulation of myocardial tissue by epinephrine, norepinephrine, and dopamine results in myocardial stunning and injury, thereby reducing cardiac contractility [2]. 

Numerous precipitating factors have been documented in the literature, with almost any significant stressor capable of triggering this response. In the case above, the contribution is likely multifactorial. COVID-19 infection may have played a role, as viral illnesses are increasingly recognized as potential triggers. Additionally, severe hyponatremia likely served as a secondary physiologic stressor. Although identifying the primary etiologic factor is challenging, recognizing these associations remains essential in patients with Takotsubo cardiomyopathy [5]. 

Multiple case reports have described COVID-19-associated Takotsubo cardiomyopathy, particularly given the recent global rise in COVID-19 prevalence. One 2023 study compared outcomes between COVID-related Takotsubo cardiomyopathy and Takotsubo cases without COVID-19 and demonstrated mortality rates of 32% versus 14%, respectively. The authors attributed this disparity to greater comorbidities, including COPD, and older patient age, frequently above 50 years. These findings further support that older individuals with COVID-associated Takotsubo cardiomyopathy are at higher risk of mortality, consistent with the demographics of the patient discussed above. Early recognition of Takotsubo cardiomyopathy, prompt identification of the precipitating stressor, and initiation of treatment when appropriate may reduce mortality associated with this syndrome [2]. 

Hyponatremia-associated Takotsubo cardiomyopathy is also well-described. In evaluating such cases, identification of the underlying cause of hyponatremia is crucial. Several reports have linked hyponatremia secondary to syndrome of inappropriate antidiuretic hormone secretion (SIADH) [6-8]. Other cases describe primary polydipsia or diuretic therapy as mechanisms leading to hyponatremia-triggered Takotsubo cardiomyopathy. In our patient, the profound hyponatremia was suspected to result from gastrointestinal losses due to vomiting, compounded by recently escalated outpatient HCTZ therapy, ultimately leading to myocardial stunning and ventricular dysfunction. 

As noted, many factors may contribute to the development of Takotsubo cardiomyopathy. Most case reports identify a single precipitant; however, in this case, both COVID-19 infection and severe hyponatremia appear to have contributed, making it uncommon among previously published reports. 

Another relevant consideration lies in the diagnostic framework for Takotsubo cardiomyopathy. The Revised Mayo Clinic Criteria, established in 2008, remain the most commonly utilized clinical tool [9]. Although widely adopted, portions of the criteria can prompt additional imaging, such as cardiac magnetic resonance, to exclude pheochromocytoma or myocarditis, potentially increasing healthcare utilization without altering clinical management. These elements should be interpreted within a clinical context. If there is no clinical suspicion for myocarditis or pheochromocytoma, a diagnosis of Takotsubo cardiomyopathy can be considered reasonable without requiring unnecessary imaging. 

Additionally, ACS should be included in the differential diagnosis, as ischemia may produce wall motion abnormalities resembling Takotsubo cardiomyopathy. To help distinguish these two entities, the InterTAK Diagnostic Score was developed. This bedside tool incorporates seven readily identifiable clinical parameters and does not require advanced imaging [10]. In a 2017 study by Ghadri et al., the scoring system demonstrated high diagnostic accuracy. A cutoff score ≥40 yielded 89% sensitivity and 91% specificity [4]. Scores ≥50 correctly identified Takotsubo cardiomyopathy in 95% of patients [4], while scores <31 diagnosed ACS correctly in 95% of cases [4]. The calculated InterTAK score for this patient was 62, with points attributed to female sex, the presence of a physical trigger, and an emotional trigger. Importantly, the InterTAK criteria acknowledge that coronary artery disease may coexist with Takotsubo cardiomyopathy and that pheochromocytoma may trigger the syndrome, highlighting nuances not reflected in the Revised Mayo Criteria [10]. 

In summary, Takotsubo cardiomyopathy has a broad spectrum of reported triggers. While diagnostic criteria remain essential, clinicians must recognize that not every element requires exhaustive testing, particularly when clinical features strongly support the diagnosis. Ultimately, additional refinement of diagnostic frameworks may reduce unnecessary healthcare expenditures while maintaining diagnostic accuracy. 

## Conclusions

Takotsubo cardiomyopathy is an important diagnostic consideration in patients presenting with acute cardiac dysfunction, particularly in the setting of physiological or infectious stress. Early identification allows timely initiation of appropriate medical therapy and ensures close cardiology follow-up to monitor recovery. Identifying the underlying trigger is essential to reducing the risk of recurrence. This case emphasizes stress-induced cardiomyopathy as a potential cardiac manifestation of COVID-19 infection, with severe hyponatremia contributing to its pathogenesis. 
